# State of Virtual Reality Based Disaster Preparedness and Response Training

**DOI:** 10.1371/currents.dis.1ea2b2e71237d5337fa53982a38b2aff

**Published:** 2013-04-24

**Authors:** Edbert B. Hsu, Yang Li, Jamil D. Bayram, David Levinson, Samuel Yang, Colleen Monahan

**Affiliations:** Department of Emergency Medicine, Johns Hopkins University and the Office of Critical Event Preparedness and Response (CEPAR), Johns Hopkins University, Baltimore, Maryland, USA; Center for Naval Analyses (CNA), Alexandria, Virginia, USA; Department of Emergency Medicine, Johns Hopkins University and the Office of Critical Event Preparedness and Response (CEPAR), Johns Hopkins University, Baltimore, Maryland, USA; Cranial Tap, Inc., Roundhill, Virginia, USA; School of Medicine, Institute of NanoBioTechnologyJohns Hopkins University; Advancement of Distance Education (CADE), University of Illinois, Chicago, Illinois, USA

## Abstract

The advent of technologically-based approaches to disaster response training through Virtual Reality (VR) environments appears promising in its ability to bridge the gaps of other commonly established training formats. Specifically, the immersive and participatory nature of VR training offers a unique realistic quality that is not generally present in classroom-based or web-based training, yet retains considerable cost advantages over large-scale real-life exercises and other modalities and is gaining increasing acceptance. Currently, numerous government departments and agencies including the U.S. Department of Homeland Security (DHS), the Centers for Disease Control and Prevention (CDC) as well as academic institutions are exploring the unique advantages of VR-based training for disaster preparedness and response. Growing implementation of VR-based training for disaster preparedness and response, conducted either independently or combined with other training formats, is anticipated. This paper reviews several applications of VR-based training in the United States, and reveals advantages as well as potential drawbacks and challenges associated with the implementation of such training platform.

## INTRODUCTION

Effective training is a cornerstone of disaster preparedness efforts worldwide. The quality, consistency and frequency of disaster training are acknowledged to notably impact self-perceived disaster readiness of potential responders.^1^ Yet, while the importance of such training is widely recognized, barriers such as time, cost and safety limit the extent to which large groups of responders can be brought up to established standards, particularly related to integrated disaster team response experience and skills. This is especially evident during events involving large-scale mobilization of public health resources and delivery of population-based healthcare where skills learned through training may directly impact actual response.

Although large-scale events like the September 11^th^ terrorist attacks in New York City and Hurricane Katrina in Louisiana (United States) have prompted additional emphasis on disaster response training and exercises, preparedness efforts continue to primarily rely on three conventional training methods: 1) classroom-based instructive teaching; 2) web-based training that consists primarily of pre-recorded, user-paced presentation material; and 3) real-life drills and tabletop exercises of varying scales. While all are long-established and instructionally valid approaches, classroom-based instructive teaching and web-based presentation material lack the realism offered by drills and exercises. At the same time, real-life drills and tabletop exercise programs are often inconsistent because of varying levels of participation or the extent of time and resources required for design, execution and review. The advent of technologically-based approaches to disaster preparedness through virtual reality (VR) environments appears promising in its ability to bridge the gaps of other commonly-held established training formats. Some government agencies have adopted VR-based applications to host meetings and a number of academic institutions and organizations have piloted VR-based training to assess program utility and effectiveness. However, applications of such systems for disaster training remain in their early stages of implementation.

Over the past decade, VR-based training in disaster preparedness has been increasingly recognized as an important and novel alternative to traditional modalities of real-life drills and table-top exercises. Several studies have described various applications of virtual reality in disaster training. In 2001, Freeman et al. published the use of a virtual reality patient simulation system for teaching emergency response skills to U.S. Navy medical providers.^2^ In 2007, a virtual simulation-enhanced triage training for Iraqi medical personnel was described.^3^ The following year, immersive simulation for training first responders for mass casualty incidents,^4^ mass casualty triage skills using immersive three-dimensional virtual reality^5^ equivalency of VR simulators with use of live actor-patients in prompting critical actions during mass casualty drills,^6^ and simulation for team training and assessment using virtual worlds^7^ were demonstrated. In 2009, game-based mass casualty burn training was proposed.^8^ In 2011, Cone et al. published a comparison of the SALT and SMART triage systems using a virtual reality simulator with paramedic students.^9^ Most recently, virtual simulation as an instructional method for nursing students was demonstrated to reinforce learning and improve learning retention over time.^10^

## SCOPE

VR-based systems encompass a wide array of technical capabilities ranging from personal computer-based software to fully immersive and high-fidelity platforms where participants don 3-D goggles in controlled environments.^2^ ^3^ ^4^ For the purposes of this paper, the scope of VR-based disaster training applications is limited to those using a personal computer and software with broadband internet connection. The ubiquity and simplicity of this interface may facilitate greater interaction among participants as compared to highly intricate VR systems that require more advanced technical capabilities and cost to operate.

## RATIONALE FOR VR-BASED TRAINING

During a disaster or public health emergency, the ability for responders to react appropriately is driven not only by pre-existing knowledge and skills, but also to a considerable degree, their psychological state of mind and familiarity with similar scenarios. Particularly during high impact, low probability events, appropriate personnel response relies upon the ability to perform their designated roles. Unforeseen psychological effects of stress brought about by unfamiliar environments or situations can impair decision-making and directly affect performance, leading to degradation of even routinely practiced skills. Disaster or public health emergency training scenarios incorporating real event elements (e.g., large crowds, damage to infrastructure, background noise, visual and auditory cues) can better approximate real life conditions while retaining the advantages of a controlled environment. This increased practice realism enables responders to gauge their individual and/or team’s ability to execute tasks and decision-making under more closely representative conditions. With continuing advancements in information technology, VR-based training that incorporates life-like realism meets these criteria and is becoming a viable alternative to conventional training. In essence, the immersive environment incorporated in VR-based training and exercise applications not only offers the realism that classroom-based instructive teaching and web-based educational material lack, but also may reduce the time and cost burden that real-life drills and tabletop exercises can place on participating individuals and sponsoring organizations.

## ADVANTAGES OF VR-BASED TRAINING

Interactive VR-based disaster training can be tailored to specific users as well as organizations, based on their resources and hazard vulnerability analysis. For example, VR-based scenarios can be developed for instructional task-focused training in which the program responds to user inputs and provides instant feedback, such as performance of mass casualty triage skills. In addition, a VR-based exercise can also allow an organization to test its emergency response plans in order to assess its effectiveness, and in turn, identify gaps and areas for improvement. Furthermore, VR-based applications can also facilitate consistent and repeated training over geographical and organizational divides. For example, VR-based applications can provide consistent synchronous multi-region and multi-organization trainings for natural occurrences such as hurricane or earthquake response. Traditional instructional elements, such as slide presentations and graphics, may readily be embedded within the VR environment but made accessible in novel ways for trainees. Another significant advantage of VR-based systems is the ability to incorporate additional realistic audio-visual stimuli, such as video clips depicting a mock event in progress or news reports that convey further information from the disaster.

Comparatively, VR-based training holds major advantages over conventional training. From an environment perspective, VR-based applications using programs such as Second Life^®^ or Open Simulator^®^ not only have the ability to incorporate life-like scenarios with avatars, but also allow reaction to user input and provide instant feedback. An “avatar” is the digital (graphical) representation of the user within the virtual reality world. For example, a skills-based Simple Triage and Rapid Treatment (START) training scenario can instantly determine if a patient was correctly assessed and provide feedback to the trainee. Virtual human characters can be programmed to act and respond to conditions set forth by the situational controls. Modules of behavioral patterns can be preselected or unfold based upon decisions made by users.

Individual VR-based applications also allow trainees to work at their own pace, thus providing the opportunity to fully grasp disaster-related concepts through asynchronous learning. Participants may interact with virtual environment components such as medical interventions, equipment, data panels, transportation options and other disaster response features. In addition, VR-based applications also provide a platform for participants to interact through text or voice communication inside the task-focused scenario, including dialogue with virtual patients and providers representing various cultural and socioeconomic demographics. Virtual environments can also be applied to groups. Through use of a control panel, actions can be set into motion providing a setting for group discussion and analysis. The scene action can be paused and variables then applied to study the effects of unexpected occurrences. This can be especially useful for exercises conducted at multiple locations simultaneously using the same scenario (e.g. a unified command disaster response exercise that involves multiple responding organizations across multiple jurisdictions). Environmental features constructed to replicate real world settings may incorporate buildings, vegetation, human effects and sound. Further, VR-based training and exercise scenarios can simulate environments located in various residential and urban settings that would be very challenging for real-life exercises. For instance, there are considerable logistical considerations to completely shut down several city blocks for the purposes of an exercise involving a terrorist incident. In contrast, VR-based programs can readily and realistically simulate these complex environments without real-life disruption.

From a cost perspective, VR-based disaster training has significant advantages. From relatively simple table-top exercises where participants convene in a conference setting for discussion, to more complex full scale exercises where personnel and equipment are mobilized, real-life drills and exercises are expensive in both time and resources required. In an era of ever-tighter fiscal constraints, the available resources and funding to support disaster training has become increasingly restricted, highlighting the need for effective, cost-conscious solutions. At the federal level, planning in recent years for extensive multi-agency exercises held concurrently in different cities have been scaled back while many state and local agencies are experiencing similar mandated reductions. VR-based disaster training offers a practical alternative that incorporates realism at a fraction of the cost of real-life exercises when considering the number of potential learners, range of applications and repeat scenario use.^11^ Accordingly, fundamental training scenarios can be practiced more frequently under different varying conditions to either challenge responders or to establish better understanding of factors that may lead to alternate outcomes.

Lastly, since VR-based scenarios and exercise play can be digitally stored, evaluators may more effectively review training and exercise conduct. VR-based platforms support data and video capture of time and critical action elements, which can be invaluable for the analysis of disaster response. This can be used to more accurately gather lessons learned and develop corrective actions necessary for the after-action review process. It should be pointed out that VR-based scenarios do not have to be used strictly independently, but rather could be combined or adapted with other traditional instructional formats to capture the distinctive advantages of each.

## POTENTIAL DRAWBACKS

Although VR-based training and exercise applications provide a practical alternative to conventional real-life disaster training, adoption of such applications are not without inherent challenges. First and foremost, the lack of familiarity with VR applications among disaster planning leadership may be a significant barrier to adopt such technology. The intuitive nature of VR-based training and its resemblance to commercial gaming platforms may lead some to perceive VR platforms as lacking credible and validated training benefit. As VR-based training gains popularity, research aimed at comparative training effectiveness may address such issues directly. Furthermore, the initial development costs associated with a VR-based training and exercise applications may be also seen as a downside since efforts required to create a realistic and interactive scenario require significant time and resources. To an extent, the cost of development scales with enhancement of immersive realism. Despite such considerations, early development costs remain a modest fraction of full-scale live exercises and may be recouped in short order.

Given the novelty of VR-based training and exercise applications, preliminary training is also required so users can effectively use new systems, since lack of familiarity with VR-based applications can initially challenge users. As previously noted, virtual reality platforms could provide participants with a higher level of realism and immersion,^12^ when compared to classroom instructions and web-based educational material. However, compared to real-life exercises, simulated scenarios still lack the direct hands-on experience and face-to-face interactions that real-life exercises provide. Existing technology may affect accessibility, stability, resolution or number of simultaneous participants. However, continued advancement in technology are expected to overcome these current limitations.

## EXAMPLES OF VR-BASED SIMULATION DISASTER TRAINING IN THE UNITED STATES

In the United States, there are several VR-based simulation disaster training projects, on the governmental, academic, and private levels. Below are few prominent examples.

***Government agencies***

Currently, numerous government departments and agencies including the U.S. Department of Homeland Security (DHS), the Centers for Disease Control and Prevention, the National Institute of Health and the National Science Foundation have begun using VR-based programs and services to host conferences. Several are beginning to realize the distinct advantages of VR-based training. The following are three prominent examples of VR-based applications in the public sector.

- ***New York City Office of Emergency Management (OEM)***-using the Advanced Disaster Management Simulator (ADMS) training system developed by Environmental Tectonics Corporation that employs artificial intelligence, the New York City OEM has built a virtual replica of New York city.^13^ The system focuses on command element simulation and allows trainees to navigate through the virtual city by means of a joystick. Trainees can communicate emergency response needs through a facilitator who guides them through decision points and objectives.
- ***Los Angeles Police Department (LAPD)*** *– *the LAPD has been using the commercially available Hydra simulation system to foster disaster-based training for its incident command officers. This system can be interlinked with other emergency operation centers around the world. With a variety of newscasts, briefings and other simulated real-time information, Hydra features immersive simulation training with video feeds that monitor decision-making processes.^14^
- ***DHS Federal Emergency Management Agency (FEMA*** *) - *Several DHS projects in development aim to align with modeling and simulation priorities at the FEMA National Exercise Simulation Center (NESC) to create tools that may support future National Level Exercises.

***Academia***

Similarly, academic institutions are exploring the unique advantages of VR-based training for disaster preparedness and response. The following are prominent examples of VR-based training applications and systems developed by academic institutions for emergency response training.

- ***The University of Illinois at Chicago School of Public Health - ***Center for Advancement of Distance Education (UIC-SPH-CADE) – UIC-SPH-CADE has been working in the well-established VR application Second Life^®^ developed by Linden Lab^®^ since 2006.^15^The model established for preparedness planning combined the use of a simulated environment coupled with facilitated discussion. Virtual environments included construction of a scale dispensing and vaccination center along with a large convention center and hospital for primary, surge and alternate care simulations. Subsequent projects included development of virtual Receiving, Staging and Storage (RSS) and drop sites to train regional preparedness coordinators in Illinois in their role to work with the Strategic National Stockpile (SNS).

**Figure:**
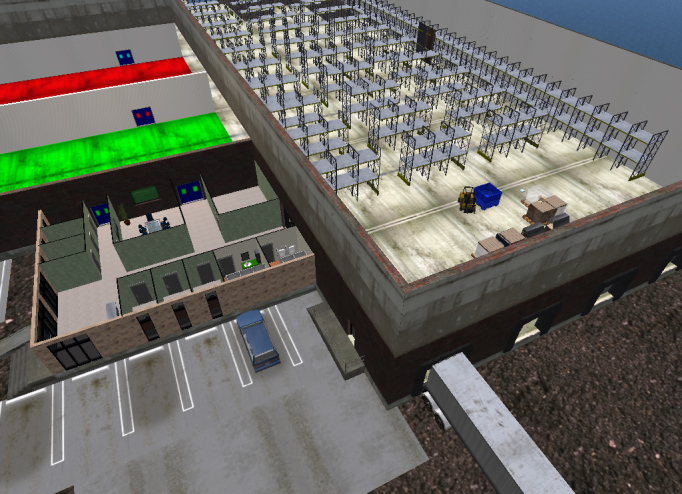
Virtual Receiving Staging and Storage (RSS)

******
- ***University*** ***of Minnesota Public Health Preparedness Center - ***An ongoing, multi-year virtual reality preparedness research project sponsored by the CDC called “Using Collaborative Virtual Environments in Preparedness and Emergency Response Planning” is led by the UIC-SPH-CADE through the University of Minnesota Public Health Preparedness Center.^16^ This observational study focusing on mass pharmaceutical dispensing uses intervention and control groups from forty local health departments nationwide and compares the VR-facilitated exercises to those performed without using VR simulation.******
- ***The Johns Hopkins Office of Critical Event Preparedness and Response (CEPAR) - ***A VR-based training under development features six disaster preparedness modules covering the topics of triage, personal protective equipment and decontamination, as well as biological, chemical and radiological threats. Located in a virtual modern hospital complex, this 3-D training environment also includes an area for facilitating tabletop exercises and a focused mass casualty triage exercise for trainees.

**Figure:**
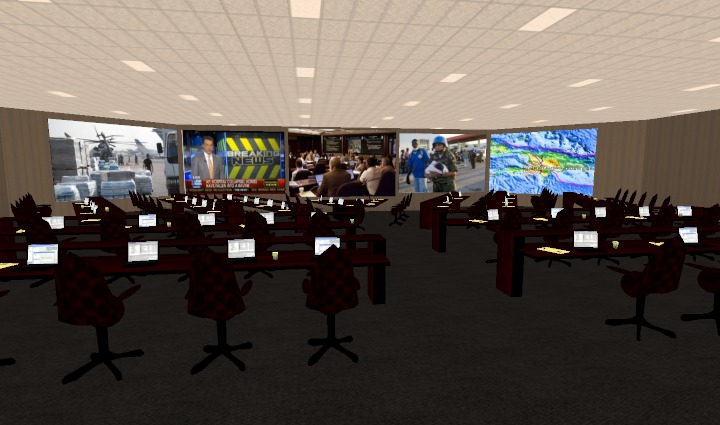
Virtual Tabletop Exercise

The ability to create characters (i.e. avatars as patients or emergency response personnel) and tailor the scenario environment to geographical, meteorological and behavioral characteristics makes Second Life^®^ideal for developing virtual disaster response training or exercise scenarios. Research is underway to further incorporate crowd dynamics and other social and behavioral modifying factors into virtual training.

**Figure:**
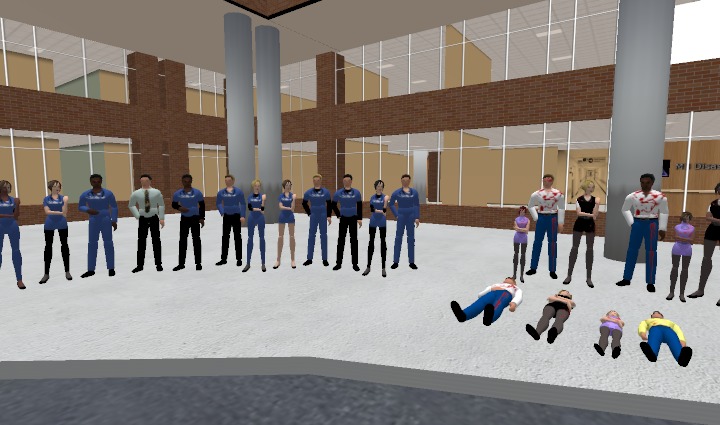
Virtual Reality Mass Casualty Triage
- ***University*** ***of Southern Mississippi*** ***- ***A project supported by the DHS Science and Technology Directorate, known as Sportevac, simulates the challenges of a stadium evacuation with thousands of avatars. Up to 70,000 agents representing stadium workers, spectators as well as first responders allow exploration of human behaviors in response to interactive threats.^17^
- ***Dartmouth*** ***Medical School Interactive Media Laboratory (IML) - ***The Dartmouth IML created an Ops-Plus for HAZMAT application that features a Virtual Terrorism Response Academy.^18^ The application includes interactive content in an immersive 3-D environment that aids trainees responding to various terrorism threats such as chemical and biological hazards. The virtual training emphasizes core competencies pertaining to risk, individual and team safety.
- ***University*** ***of Florida-*** In collaboration with 360ed, Inc., University of Florida produced “Burn Center”; a web-based virtual reality simulation game created to train and prepare healthcare providers in the treatment of burn injuries. It provides instruction in the management of mass casualty burn victims, both at the scene in a fast-paced triage scenario and in a hospital setting during the first 36 hours of post-injury resuscitation care. Trainees earn continuing medical education credits upon completing the training.^19^

## CONCLUSION

The emergence of virtual reality platform-based technologies applied to disaster preparedness and response training offers significant potential advantages over other traditional forms of training, and is gaining increasing acceptance. The immersive and participatory nature of VR training offers a unique realistic quality to training that is not generally present in classroom-based or web-based modalities, yet retains considerable cost advantages over large-scale real-life exercises. Growing implementation of VR-based training for disaster preparedness and response, conducted either independently or combined with other training formats, to realize these distinct benefits is a keenly anticipated development. Comparative research between VR-based and traditional modalities of disaster training is needed to explore the various aspects of realism, cost, and ultimately disaster readiness.

## Competing Interests

The authors have declared that no competing interests exist.
